# Magnetic Iron Oxide Nanoneedles with Hierarchical Structure for Controllable Catalytic Activity of 4-Nitrophenol Reduction

**DOI:** 10.3390/nano13061037

**Published:** 2023-03-13

**Authors:** Hyokyung Jeon, Ha-Jin Lee

**Affiliations:** 1 Western Seoul Center, Korea Basic Science Institute, 150 Bugahyun-ro, Seoudaemun-gu, Seoul 03759, Republic of Korea; 2 Division of Chemistry and Bio-Environmental Sciences, Seoul Women’s University, 621 Hwarangro, Nowon-gu, Seoul 01797, Republic of Korea

**Keywords:** magnetic nanoparticle, polydopamine, multi-metallic nanoparticles, iron oxide nanoneedles, hierarchical nanostructure, catalytic activity

## Abstract

Catalyst systems with high catalytic activity and sustainability are highly desirable. Here, we report a design for catalytic composites with a hierarchical structure in which polydopamine (PD), multi-metallic nanocatalysts and iron oxide nanoneedles are successively deposited on a magnetic core. PD layers with various thicknesses are coated onto the magnetic core and serve as a template by which to take up multi-metallic nanocatalysts such as Au, Ag and Pt nanoparticles. The iron oxide nanoneedles act as spacers, preventing the nanocomposite from aggregating and increasing the surface area of the composite. The distinctive structures of the controllable template, the multi-metallic catalysts and needle-like layers enable the rapid migration of reactive ionic species and enhance catalytic ability via the synergistic effect of the multi-metallic nanocatalysts and iron oxide nanoneedles. Moreover, due to the strong magnetic property of the catalytic nanocomposites, they can be easily recovered with an external magnet and reused. Our hierarchical nanocomposites for recyclable nanocatalysts provide a new design concept for highly efficient catalysts.

## 1. Introduction

Novel metal nanoparticles are of great interest in the development of highly efficient catalysts for the treatment of pollutants because they have a high specific surface area that favors the interaction of reactants with the surface of catalysts [1,2,3,4,5,6]. However, despite their outstanding catalytic activity, metal nanoparticles have technical limitations. For instance, decreasing the size of particles to the nanoscale can increase their surface area and surface energy, which can lead to instability in an aqueous solution and aggregation into large particles [7,8]. As a result, their catalytic efficiency is eventually decreased. To address this problem, metal nanoparticles have been introduced to mediate materials, such as polymers, carbon nanostructures and other metal oxides [9,10,11,12].

Polydopamine (PD), which was inspired by mussel adhesive proteins, has been extensively researched as one of the simplest and most versatile strategies for surface functionalization [13,14]. PD exhibits good biocompatibility and stability and is readily deposited on various types of surfaces via the self-polymerization of dopamine under mild alkaline conditions. PD comprises various functional groups, such as catechol, amines and imines, which can serve as reaction sites and anchors to take up metal ions [15,16,17]. Moreover, the thickness of the PD shell on templates can be controlled by varying the dopamine concentration to enrich the reaction sites and to enable the anchors to take up metal ions [16].

The design and construction of hierarchically structured micro-/nanomaterials has received considerable attention, since it enables the development of materials that have enhanced properties [18,19,20,21]. The introduction of hierarchical micro/nanostructures is preferable because they can provide an extremely high surface area while retaining an interconnected network to ensure excellent charge carrier conduction [21]. In addition, a hierarchical structure exhibits unique properties that are absent from single-component materials, and these properties can be tuned by varying the structures of the hierarchical materials. Furthermore, if required, various properties can be incorporated into a single component [22,23,24,25].

The present study focuses on the design of catalytic composites with a hierarchical structure in which PD, nanocatalysts and iron oxide nanoneedles are successively deposited on a magnetic core. PD layers are coated on the magnetic nanoparticles via the self-polymerization of dopamine, which is a monomer, and the thickness of the PD layers is controlled by varying the concentration of the monomer, serving as a template on which to load noble metal nanoparticles such as Au, Ag and Pt. The iron oxide nanoneedles act as spacers, preventing the nanocomposite from aggregating and increasing the surface area of the composite. Herein, we report a novel strategy for synthesizing magnetic hierarchical nanocomposites with high catalytic activity and recyclability, and systematically study the effect of each layer structure on catalytic efficiency.

## 2. Experimental Section

### 2.1. Materials

Iron(III) chloride hexahydrate (FeCl_3_·6H_2_O, 98%), sodium acetate (C_2_H_3_NaO_2_, 99%), ethylene glycol (C_2_H_6_O_2_, 99.9%), dopamine hydrochloride, ammonium hydroxide solution (NH_4_OH), gold chloride trihydrate (HAuCl_4_·3H_2_O), silver nitrate (AgNO_3_), chloroplatinic acid hydrate (H_2_PtCl_6_·xH_2_O), sodium borohydride (NaBH_4_), iron(II) sulfate heptahydrate **(**FeSO_4_·7H_2_O), iron(III) sulfate hydrate (Fe_2_(SO_4_)_3_·xH_2_O) and 4-nitrophenol sodium salt dehydrate (C_6_H_8_NO_5_Na) were purchase from Sigma-Aldrich. All chemicals were of analytical grade and used without further purification. Deionized water with a resistivity greater than 18.2 MΩ cm was obtained using a Millipore Simplicity 185 system (Merck Millipore, Burlington, MA, USA).

### 2.2. Synthesis

*Fe_3_O_4_ magnetic nanoparticles (MNPs)*

FeCl_3_·6H_2_O (0.9 g) and sodium acetate (2.4 g) were dispersed in 30 mL of ethylene glycol under vigorous stirring for 30 min at room temperature. Subsequently, the mixture was sealed in a Teflon-lined autoclave bomb and heated at 200 °C in a drying oven for 8 h. Once the solution had cooled to room temperature, the black MNPs were obtained via centrifugation and washed in ethanol and deionized (DI) water three times.

*Polydopamine-coated MNPs (MNP@PD_x_; x = 30, 60 and 100)*

The as-prepared MNPs (200 mg, for x = 30) were dispersed in a mixed solution of DI water (9 mL), ethanol (4 mL) and NH_4_OH solution (0.04 mL) and underwent mechanical stirring for 30 min at room temperature. Here, x represents the thickness (nm) of the polydopamine shell of MNP@PD_x_ covered by the MNP surface after dopamine polymerization. An aqueous solution (1 mL) containing dopamine hydrochloride (50 mg for x = 30) was added to the MNP solution and was stirred for 30 h. The final black product, MNP@PD_30_, was collected using an external magnet and washed in DI water three times and then air dried. MNP@PD_60_ and MNP@PD_100_, in which the thicknesses of the PD layers were 60 nm and 100 nm, were obtained using MNPs (100 mg) with 50 mg and 200 mg of dopamine hydrochloride, respectively, using the same synthetic procedure as that used for MNP@PD_30_.

*Metallic nanocatalyst-coated MNP@PD (MNP@PD/M, M = Au, Au-Ag, Ag-Pt and Au-Ag-Pt)*

An aqueous solution of HAuCl_4_·3H_2_O (2.5 mM, 10 mL) was added to an aqueous suspension of MNP@PD (15 mg, 10 mL). The dispersion was vigorously agitated in a shaker for 30 min to allow the Au ions to adsorb onto the polydopamine layer. The Au ions were taken up by the MNP@PD and were reduced via treatment with an NaBH_4_ solution (5 mM, 1 mL) for 5 min. The Au nanocatalyst-coated MNP@PD/Au was collected with an external magnet and washed three times in DI water. Bi-metallic catalysts, MNP@PD/Au-Ag and MNP@PD/Au-Ag, were obtained by mixing AgNO_3_ (2.5 mM, 5 mL) or H_2_PtCl_6_·xH_2_O (2.5 mM, 5 mL) with HAuCl_4_·3H_2_O (2.5 mM, 5 mL). Similarly, the tri-metallic MNP@PD/Au-Ag-Pt catalysts were obtained by mixing AgNO_3_ (2.5 mM, 2.5 mL), H_2_PtCl_6_·xH_2_O (2.5 mM, 2.5 mL) and HAuCl_4_·3H_2_O (2.5 mM, 5 mL).

*Iron oxide nanoneedle-coated MNP@PD/M (MNP@PD/M/IO).*

An aqueous solution (7.5 mL) containing FeSO_4_ (1.5 mM) and Fe_2_(SO_4_)_3_ (2.0 mM) was added to a suspension of MNP@PD/M (10 mg, 10 mL). The dispersion was vigorously agitated in a shaker for 3 h to allow the Fe ions to adsorb onto MNP@PD/M. The Fe ions that adsorbed onto MNP@PD/M were hydrolyzed by the oxygen in the air and in the solution to form iron oxide [22]. The iron-oxide-coated MNP@PD/M (MNP@PD/M/IO) was then collected using an external magnet and washed three times with DI water.

### 2.3. Catalytic Test

The catalytic reduction of 4-nitrophenol (4-NPh) to 4-aminophenol (4-APh) using hierarchical nanocatalysts was studied via UV–vis absorption spectroscopy. A NaBH_4_ aqueous solution (10 mM, 1.5 mL) and 4-NPh aqueous solution (0.3 mM, 1.0 mL) were added to a UV quartz cell (1 × 1 cm^2^), and 0.2 mL of each nanocatalyst (1 mg/mL) was added to the mixture cell. The absorption spectra were recorded at regular intervals in the spectral range 200–600 nm, and the concentration of 4-NPh in the mixture was determined at 400 nm in the absorption spectra. For the recycling test, the nanocatalysts were magnetically separated from the mixture, washed three times in DI water and then reused.

### 2.4. Characterization

The morphologies of the catalytic nanocomposites were characterized using FE-TEM/STEM/EDX (field emission–transmission electron microscopy/scanning transmission electron microscopy/energy-dispersive X-ray) and FE-SEM (field emission–scanning electron microscopy) using a JEOL microscope (JEM-2200 FS, Tokyo, Japan) operated at 200 kV and a Hitachi S-4700 microscope, respectively, at the Jeonju Center of the Korea Basic Science Institute (KBSI). Powder X-ray diffraction (XRD) analysis was carried out on a Panalytical X’Pert Pro MPD diffractometer (Malvern, UK) using Cu Kα radiation at the Western Seoul Center of KBSI. X-ray photoelectron spectroscopy (XPS) studies were performed using a K-ALPHA+ (Thermo Scientific, Waltham, MA, USA) system with an aluminum anode (Al K*α*, 1486.6 eV) at 12 kV and at 72 W at the Busan Center of KBSI. The magnetic properties of the samples were obtained using a vibrating sample magnetometer (MPMS@3 SQUID-VSM, Quantum Design, San Diego, CA, USA) at the KBSI, and magnetic performance over a range from −20 to +20 kOe was recorded at 300 K. Fourier-transform infrared (FT-IR) measurements were recorded using the KBr pellet method on a Nicolet iS10 (ThermoFisher Scientific, Waltham, MA, USA) in the range 4000~500 cm^−1^. TGA was performed using a thermogravimetric analyzer (Scinco TGA N-1500, Seoul, Korea) over the temperature range 25~800 °C at a heating rate of 10 °C min^−1^ under air (flow rate, 60 cm^2^ min^−1^). The UV-vis absorption spectra were recorded on a UV-vis-NIR spectrophotometer (Shimadzu UV-3600, Kyoto, Japan).

## 3. Results and Discussion

### 3.1. Structure Characterization of Magnetic Hierarchical Nanocomposites

**Figure 1:**
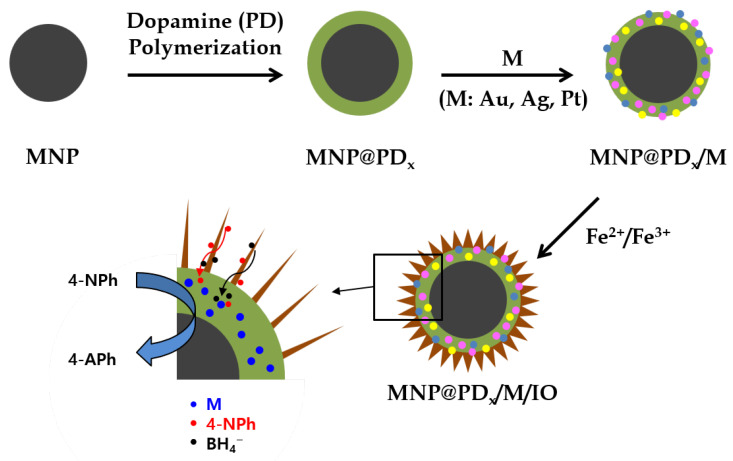
Schematic of the procedure for the preparation of iron oxide nanoneedles with a hierarchical structure by successively depositing layers of polydopamine (PD), metal nanoparticles (M) and iron oxide (IO) on the magnetic nanoparticles (MNPs) and catalytic application for 4-NPh reduction. x represents the thickness of the PD layer in nm (x = 30, 60 and 100).

Figure 1 shows a schematic illustration of the construction of magnetic hierarchical nanocomposites. Magnetic core nanoparticles (MNP) of Fe_3_O_4_ with a ~400 nm diameter were obtained via a solvothermal method at 200 °C by carrying out the reduction of FeCl_3_ with ethylene glycol under alkali conditions. Ethylene glycol is a good reducing agent that is widely used in the polyol process to prepare metal oxide nanoparticles [26,27]. Sodium acetate was added as an alkali source and electrostatic stabilizer [28].

**Figure 2:**
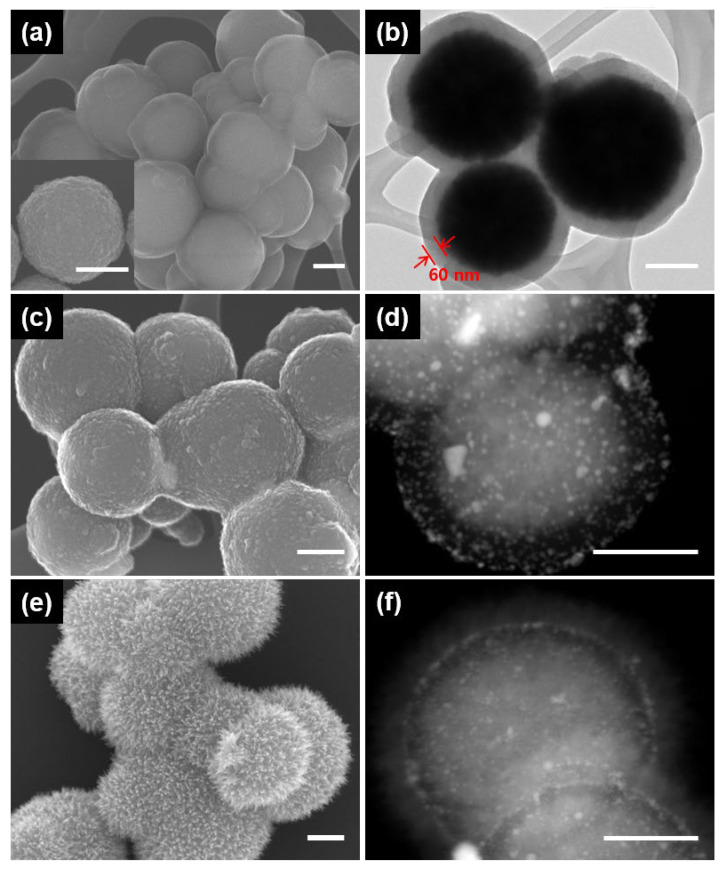
SEM and STEM images showing the formation of the magnetic hierarchical nanocomposites; (**a**,**b**) MNP@PD_60_; (**c**,**d**) MNP@PD_60_/Au; and (**e**,**f**) MNP@PD_60_/Au/IO. Inset of (**a**) is an SEM image of the magnetic nanoparticles (MNPs). All scale bars represent 200 nm.

Figure 2 shows the SEM and TEM images of the magnetic hierarchical nanocomposites that were successively deposited onto MNPs by polydopamine (PD), metal nanoparticles and iron oxide nanoneedles. The MNPs were first coated with PD through the spontaneous self-polymerization of dopamine in an alkaline medium, resulting in the formation of a core@shell structure (MNP@PD). The SEM and STEM images of MNP@PD in Figure 2a,b clearly show that the MNP core is uniformly encapsulated by a PD layer with a thickness of 60 nm when an MNP to dopamine mass ratio of 1:0.5 was used. The thickness of the PD layer was controlled by changing the mass between MNPs and dopamine. When the mass ratio was changed to 1:0.25 and 1:2, it was confirmed that the thicknesses of the polydopamine layer were 30 nm and 100 nm, respectively (Figure S1).

Figure S2 presents the FTIR spectra and TGA data of MNP@PD. In Figure S2a, the broad and weak peaks around ~3440 cm^−1^ correspond to the stretching vibration of water adsorbed on the surface of the hydroxyl groups of PD. The peaks at 1620 cm^−1^, 1475 cm^−1^ and 1350 cm^−1^ were assigned to the stretching vibrations of the C=C, C-O and C-N bonds from the PD layer, respectively [29]. The strong peak around 630 cm^−1^ is related to the Fe-O vibration of the MNP core [30]. As the thickness of the PD layer increases, the overall peak intensity corresponding to the organic functional groups (1600~1250 cm^−1^, orange region) increases compared to the Fe-O groups (630 cm^−1^, blue region). This result indicates the successful use of PD layers with different thickness values on the MNP core achieved by controlling the amount of dopamine. The TGA results further support this by showing the evident weight loss at 800 °C, increasing from 19.5% (for MNP@PD_30_) to 38.3% (for MNP@PD_100_) (Figure S2b).

The Au nanoparticles (AuNPs) used as nanocatalysts were synthesized on MNP@PD via the absorption of HAuCl_4_ and reduction of the NaBH_4_ reducing agent (Figure 2c,d, denoted as MNP@PD/Au). The catechol and amine functional groups of the PD layer served as binding sites on which to take up the AuCl_4_^−^ metal ion precursor [31,32]. The surface morphology of MNP@PD appeared to be slightly rough after the formation of MNP@PD/Au compared with the formation of MNP@PD due to the formation of AuNPs (Figure 2a,c). The STEM images and the corresponding EDX data confirm the presence of AuNPs on MNP@PD particles (Figure 2c and Figure S3a). Iron oxide (IO) nanoneedles were grown on the surface of MNP@PD/Au via the addition of a mixed aqueous precursor solution of Fe^2+^/Fe^3+^ and the controlled oxidation of the iron precursors under ambient conditions (Figure 2e,f). The iron precursors were hydrolyzed by the oxygen in the solution to form needle-like iron oxide nanoparticles [22,33]. MNP@PD/Au particles were covered with needle-like IO nanocrystals, and the relative atomic content of Fe in the IO nanoneedle-coated MNP (MNP@PD/Au/IO) increased compared with that of MNP@PD/Au (Figure S3b).

**Figure 3:**
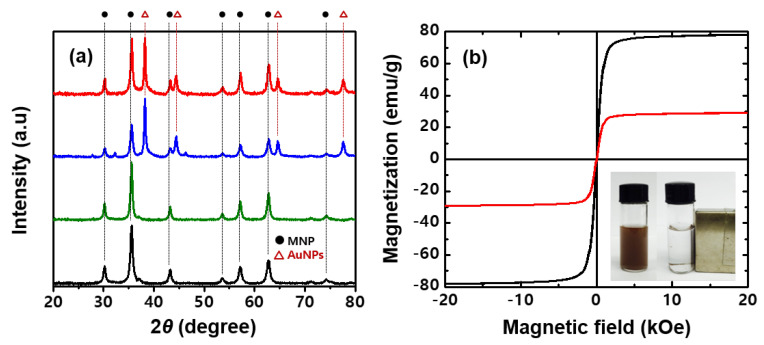
(**a**) XRD patterns of MNPs (black line), MNP@PD (green line), MNP@PD/Au (blue line) and MNP@PD/Au/IO (red line). (**b**) Field-dependent magnetic curves of MNPs (black line) and MNP@PD/Au/IO (red line). Inset of (**b**) is a photograph of MNP@PD/Au/IO dispersed in water (**left**) and attracted to the side of the container after applying an external magnetic field (**right**).

The X-ray diffraction (XRD) patterns of MNP, MNP@PD, MNP@PD/Au and MNP@PD/Au/IO are shown in Figure 3a. Seven characteristic peaks at 2*θ* = 30.2°, 35.5°, 43.2°, 53.6°, 57.1°, 62.7° and 74.2° correspond to the (220), (311), (400), (422), (511), (440) and (533) planes, respectively, which represent a face-centered cubic phase of Fe_3_O_4_ (ICSD card no. 01-075-0033) [34,35]. These crystalline phases of MNP did not change after successive coating with PD, the metal nanoparticles and IO, which means that the hierarchical coating did not affect the crystal structure of MNP. The XRD peaks of MNP@PD/Au and MNP@PD/Au/IO were identified at 38.2°, 44.4°, 64.6° and 77.5°, which correspond to the (111), (220), (220) and (311) lattice planes of the cubic phase of Au (ICSD card no. 01-089-3697) [36]. In addition, the characteristic IO peaks on the MNP@PD/Au composites did not appear due to low crystallinity. Figure 3b shows the hysteresis loops of the MNP and MNP@PD/Au/IO composites examined using a vibrating sample magnetometer (VSM) at 300 K. Both particles exhibit characteristic S-shaped curves for the magnetic moment (*M*) versus the magnetic field (*H*), corresponding to typical superparamagnetic behavior, in which no obvious coercivity or remanence exists. The saturation magnetization (*M*s) of MNP was 78 emu/g, and after the formation of the hierarchical MNP@PD/Au/IO structure, it decreased to 29 emu/g. Due to the sufficient saturation magnetization, all of the hierarchical catalytic composites were isolated from the solution with external magnets after the completion of synthesis and the catalytic reaction.

Moreover, multi-metallic hybrid NPs generally show higher catalytic activity in comparison to mono-metallic NPs due to a synergetic effect [22,37,38,39,40,41]. For this purpose, bi- and tri-metallic NPs (Au-Ag, Au-Pt and Au-Ag-Pt) were introduced to the MNP@PD particles. Figure S4 displays STEM images and the corresponding EDX spectra of MNP@PD-attached bi-metallic (Au-Ag and Au-Pt) and tri-metallic NPs (Au-Ag-Pt). Small particles that are less than 10 nm in size can be observed in the PD layers, and the EDS data confirm the successful incorporation of the bi- and tri-metallic nanoparticles onto the MNP@PD particles. The multi-metallic NP embedded MNP@PD particles were further treated to grow IO nanoneedles on the surfaces of the MNP@PD particles.

**Figure 4:**
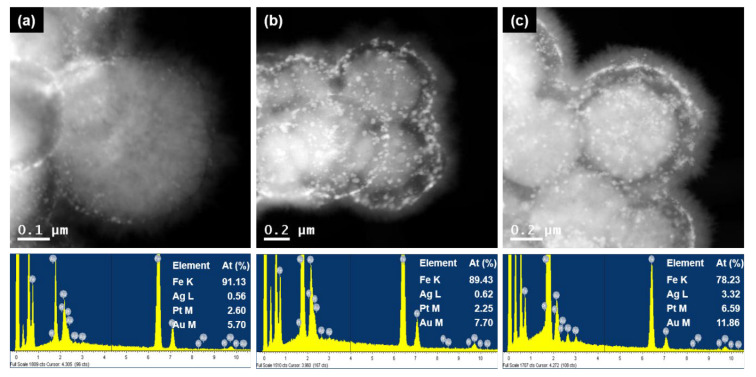
STEM images and the corresponding EDX data of MNP@PD_x_/Au-Ag-Pt/IO, where x is (**a**) 30, (**b**) 60 and (**c**) 100.

Figure 4 and Figure S5 show STEM images and the corresponding EDX data of the MNP@PD/M particles, here identified as M = Au-Ag, Au-Pt and Au-Ag-Pt. It can be observed that all of the MNP@PD/M/IO particles were densely covered with needle-like IO nanocrystals. It should be noted that the ratio of the total amount of metal nanocatalysts (M) to the amount of Fe is similar (Fe:M = 90:10) for all three MNP@PD/M/IO particles, in which the thickness of the PD layer of each compound is the same (Figure S5). STEM images and the corresponding EDX data of MNP@PDx/Au-Ag-Pt/IO particles with PD layer structures of varying thicknesses and tri-metallic NPs are shown in Figure 4. It can be observed that the ratio of the total amount of metal nanocatalysts to the amount of Fe increases as the thickness of the PD layer increases from 9% (for x = 30) to 12% (for x = 100). This is because the relatively thick PD layer possesses more functional groups (catechol, amine, hydroxyl, etc.) that can take up metal precursor ions such as AuCl_4_^−^, Ag^+^ and PtCl_6_^2−^.

### 3.2. Catalytic Activity of Magnetic Hierarchical Nanocomposites

The catalytic performances of the magnetic hierarchical nanocomposites were evaluated during the reduction of 4-nitrophenol (4-NPh), which is a water contaminant that is discharged from industrial and agricultural wastewater [42]. The reduction of 4-NPh to 4-aminophenol (4-APh) is commonly carried out in an aqueous solution using sodium borohydride (NaBH_4_) as a reducing agent and a metal catalyst as an electron transfer agent [43,44,45,46]. 4-NPh is deprotonated by NaBH_4_ to form the 4-nitorphenolate anion (4-NPh^−^), which can further react to form 4-APh. Even though the reduction of 4-NPh by NaBH_4_ is thermodynamically favorable, the reaction does not occur in the absence of a catalyst due to a mutual repellency between 4NPh^−^ and BH_4_^−^ [47]. In this study, the catalytic activity of the various magnetic hierarchical nanocomposites for the 4-NPh reduction in the presence of NaBH_4_ was examined, as was the effect of the structure on catalytic performance.

**Figure 5:**
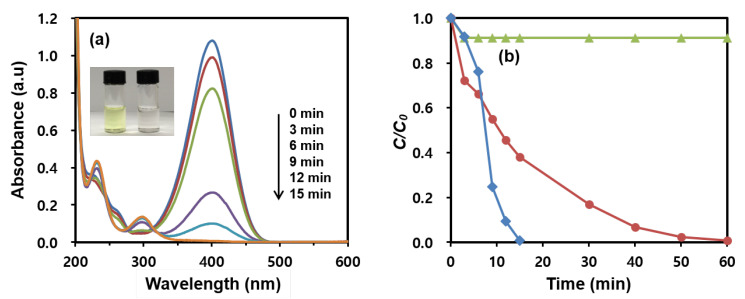
(**a**) Time-dependent UV-vis absorption spectral change in the 4-NPh reduction in the presence of MNP@PD_60_/Au/IO. Inset of (**a**) shows the color changes in the 4-NPh solution before (**left**) and after (**right**) the reduction. (**b**) Catalytic reduction rates of 4-NPh to 4-APh by MNP@PD_60_/Au/IO (red line), MNP@PD_60_/Au (blue line) and MNP@PD_60_/IO (green line).

Figure 5a shows the time-dependent spectral change in the UV-vis absorption of the 4-NPh reduction by NaBH_4_ in the presence of the MNP@PD_60_/Au/IO catalysts. Following the addition of the catalysts, a decrease in the 4-NPh absorbance at 400 nm is observed, as is an increase in the peaks of 4-APh at 230 and 300 nm with time [36,47]. The complete conversion of 4-NPh can be visually appreciated by the color change from yellow to colorless in the solution (inset in Figure 5a). Figure 5b exhibits the conversion rates of 4-NPh reduced using MNP@PD_60_/Au with and without surfaces coated with IO nanoneedles. It can be observed that the growth of IO nanoneedles on MNP@PD/Au significantly enhanced the reduction rate of 4-NPh compared to MNP@PD/Au without IO nanoneedles. In the case of MNP@PD_60_/Au, the reduction of 4-NPh was completed in 60 min. However, MNP@PD_60_/Au/IO was finished in 15 min.

To investigate the catalytic ability of the IO itself, IO nanoneedles were grown on MNP@PD_60_ without AuNPs (MNP@PD_60_/IO), and their catalytic performance was examined under the same reaction conditions. The surface morphology of MNP@PD_60_/IO is also almost the same as that of MNP@PD_60_/Au/IO, which has a densely coated nanoneedle surface structure (Figure S6). Less than 10% of the catalytic properties of MNP@PD_60_/IO without AuNPs were revealed in 60 min, indicating the almost negligible catalytic activity of the IO nanoneedles. It can be explained that due to the electron-enriched group present in iron oxides, the metal hydride complex could be formed more easily. That is, the electrons transferred from iron oxide to metal resulted in a higher electron population on metal, which facilitates the catalytic properties for the reduction reaction [2]. Hence, the existence of the electron-enriched group of iron oxides could significantly enhance the catalytic properties required for the reduction [4]. In addition, the structure of the needle shape has an advantage in increasing the catalytic efficiency. The needle-like structure of IO serves as a spacer to maintain a certain distance between the catalytic particles to prevent aggregation of the nanocatalysts [22] and allows reactants such as 4-NPh and BH_4_^−^ to easily diffuse into the catalytic particles via a capillary effect through the needle structure [48].

**Figure 6:**
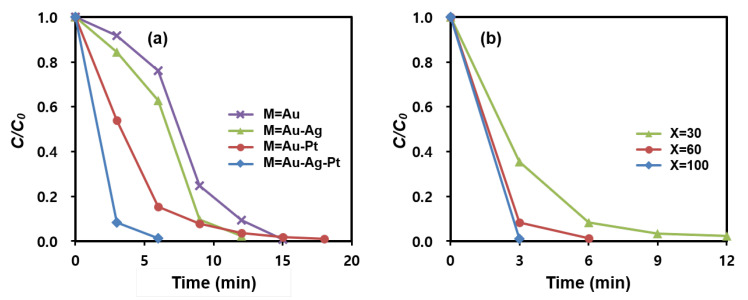
Catalytic reduction rates of 4-NPh to 4-APh by (**a**) MNP@PD_60_/M/IO (M = Au, Au-Ag, Au-Pt, and Au-Ag-Pt) and (**b**) MNP@PD_x_/Au-Ag-Pt/IO (x = 30, 60, and 100).

Figure 6a and Figure S7 display the effect of multi-metallic catalysts on the 4-NPh reduction rate. For the test, di-metallic and tri-metallic MNP@PD_60_/M/IO nanocomposites (where M is Au-Ag, Au-Pt and Au-Ag-Pt) were employed. The conversion rate of MNP@PD_60_/Au-Ag-Pt/IO was 99% after 6 min, which is higher than that of MNP@PD_60_/Au/IO (23.8%), MNP@PD_60_/Au-Ag/IO (37.2%) and MNP@PD_60_/Au-Pt/IO (84.5%). Furthermore, 15 min and 12 min were required to achieve a 99% conversion rate for MNP@PD_60_/Au/IO and MNP@PD_60_/Au-Ag (or Au-Pt)/IO, respectively. Here, it is noteworthy that the ratio of the total amount of metal nanocatalysts (M) to the amount of Fe is close to 90:10 (Fe:M) for both the bi- and tri-metallic MNP@PD_60_/M/IO nanocomposites, as shown in the results obtained for the EDX data (Figure S5). These results indicate that the enhanced catalytic performance of the multi-metallic nanocomposites can be attributed to a synergistic effect between them. In general, multi-metallic nanoparticles have outstanding catalytic properties, indicating synergistic effects between them during catalysis [37,38,39,40]. According to reports, BH_4_^−^ preferentially adheres to Pt atoms, whereas 4-NPh is more likely to adhere to Au atoms in multi-metal systems. Furthermore, excess electrons present on Au atoms can migrate to the adjacent Pt atoms [49]. Therefore, the synergistic effect in the multi-metallic nanocomposites can be explained as follows: (1) 4-NPh and BH_4_^−^ easily diffuse and adsorb onto neighboring Au, Ag and Pt atoms through the needle structure of IO by assisting in a capillary effect; and (2) the electron enrichment onto Pt can accelerate the electron transfer from BH_4_^−^ to 4-NPh on Au due to the electron transfer from Au to Pt [49,50].

MNP@PD/Au-Ag-Pt/IO particles with various PD layer thicknesses were tested for the catalytic reduction of 4-NPh, and the results are depicted in Figure 6b and Figure S8. Interestingly, the MNP@PDx/Au-Ag-Pt/IO particles with the thinnest PD layer (x = 30) and the lowest content of metal nanocatalysts (M) exhibited the highest catalytic efficiency, leading to the complete reduction of 4-NPh in 3 min. In addition, the reduction rate decreased as the thickness of the PD layer increased. According to the results of the EDX analysis, the MNP@PD_x_/Au-Ag-Pt/IO particles with a thinner PD layer had a smaller atomic percentage of metal nanocatalysts (M) (Figure 4). These results indicate that the catalytic performance of the MNP@PDx/M/IO nanocomposites is influenced by the accessibility of the reactants 4-NPh and BH_4_^−^ to the catalytic species (Au, Au and Pt NPs) rather than by the quantity of nanocatalysts present in the nanocomposites. That is, a thinner PD layer provides a shorter pathway for the molecules of the reactant 4-NPh and BH_4_^−^ to diffuse and approach the M inside the PD layer to form 4-NPh…M or BH_4_^−^…M hybrid complexes, increasing the catalytic reaction rate. This supports the explanation that catalytic efficiency can be improved by the increasing the approach rate of the reactants into the nanocatalysts (M) via a capillary effect of the IO nanoneedle structure. Typically, catalysts based on noble metal nanoparticles such as Au, Ag, and Pt exhibit high catalytic efficiency in chemical reactions, but are often costly. Nonetheless, the results of this study show that structural characteristics controlling the diffusion of molecules can enable high catalytic efficiency even with a small amount of metal nanoparticles. This effective strategy can reduce the time and costs associated with wastewater treatment.

**Figure 7:**
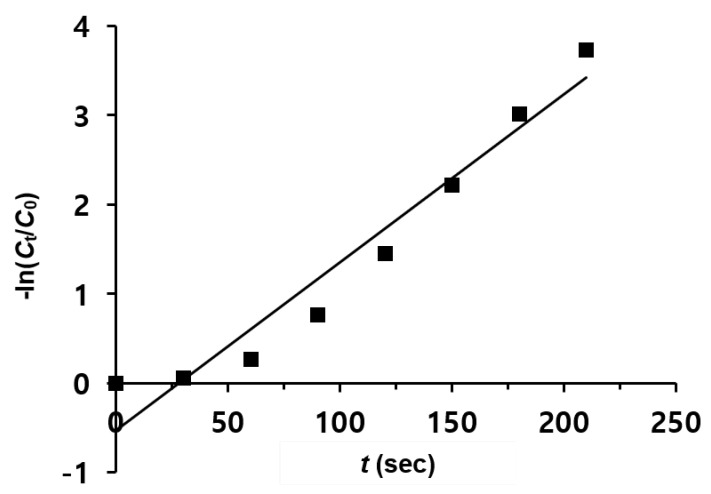
Plot of -ln(*C*_t_/*C*_0_) versus time (*t*) for the reduction of 4-NPh with MNP@PD_30_/Au-Ag-Pt/IO.

Figure 7 shows the time-dependent logarithm plot of the reduction of 4-NPh over MNP@PD_30_/Au-Ag-Pt/IO particles. Since the concentration of NaBH_4_ largely exceeds the concentration of 4-NPh (*C*_NaBH4_/*C*_4-NPh_ = 100), the reduction rate can be assumed to be independent of the BH_4_^−^ concentration [51,52]. Therefore, pseudo-first-order reaction kinetics were applied to determine the reaction rate constant. According to the slope calculated using a linear fitting relationship between −ln(*Ct*/*C*_0_) and *t*, the apparent rate constant *k_app_* was determined to be 18.8 × 10^−3^ s^−1^, which is higher than that obtained for MNP@PD_60_/Au-Ag-Pt/IO (11.9×10^−3^ s^−1^) and MNP@PD_100_/Au-Ag-Pt/IO (5.4×10^−3^ s^−1^) (Figure S9). The kinetic rate constants for the 4-NPh reduction were compared to those obtained for other catalysts, with an Fe_3_O_4_ core as the core template (Table 1). It can be clearly observed that MNP@PD/Au-Ag-Pt/IO nanocomposites have a higher kinetic rate constant, even with a lower concentration of catalysts (~0.2 mg) or the NaBH_4_ reducing agent (0.01 M).

**Figure 8:**
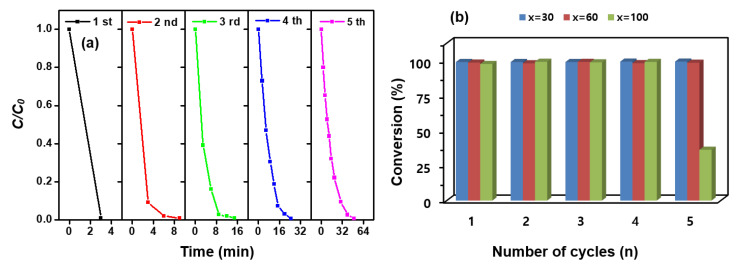
Catalytic reduction rates of 4-NPh to 4-APh as a function of the n reduction cycles by (**a**) MNP@PD_30_/Au-Ag-Pt/IO, Au-Ag-Pt) and (**b**) MNP@PD_x_/Au-Ag-Pt/IO (x = 30, 60 and 100).

Economically, the reuse of catalysts is important. Therefore, the reusability of MNP@PD_x_/Au-Ag-Pt/IO (x = 30, 60 and 100) was investigated by plotting the conversion rate of 4-NPh as a function of the number of reaction cycles, as shown in Figure 8a and Figure S10. The catalysts were washed with DI water under ultrasonic treatment three times and then reused for the next cycle. Although the conversion rate decreased slightly as the number of reuses increased, the conversion efficiency was still greater than 98%, except in MNP@PD_100_/Au-Ag-Pt/IO (Figure 8b).

## 4. Conclusions

Magnetic nanocomposites with a hierarchical structure were prepared by successively depositing layers of polydopamine, multi-metallic nanocatalysts and iron oxide nanoneedles onto the magnetic core. The thickness of the PD layers deposited on the magnetic core was controlled by varying the concentration of the dopamine monomer, and the PD layers served as a template by which to take up active catalytic nanoparticles such as Au, Ag and Pt as a multi-metallic system. In the outermost layer, the iron oxide nanoneedles acted as spacers, preventing the nanocomposite from aggregating and increasing the surface area of the composite. The prepared magnetic hierarchical nanocomposites were used as catalysts for the reduction of 4-nitrophenol in the presence of the NaBH_4_ reducing agent. The thinner PD layer and IO nanoneedle structure facilitated the access of the reactive species 4-NPh and BH_4_^−^ to the nanocatalysts, resulting in an improved catalytic rate compared to the catalytic nanocomposites with a thicker PD layer or without IO nanoneedles. The catalytic efficiency of the catalytic nanocomposites containing multi-metallic nanocatalysts was further improved over that of the mono-metallic composites due to the synergistic effect of the multi-metallic nanoparticles. Moreover, by taking advantage of the magnetic property, the catalytic nanocomposites were readily isolated using an external magnet and were reused up to five times without losing the catalytic efficiency. Our hierarchical nanocomposites for recyclable nanocatalysts offer a novel design concept for highly efficient catalysts.

## Figures and Tables

**Table 1:** Comparison of the catalytic activity of various metal nanocatalysts deposited on Fe_3_O_4_ nanoparticles for the reduction of 4-NPh.

Catalysts	Conc. of 4-NPh	Dose of Catalyst	Conc. of NaBH_4_	*k_app_* (× 10^−3^ s^−1^)	Time (min)	Reference
MNP@PD_30_/Au-Ag-Pt/IO	3 × 10^−4^ M	0.2 mg	1 × 10^−2^ M	18.8	3.5	this work
MNP@PD_60_/Au-Ag-Pt/IO	3 × 10^−4^ M	0.2 mg	1 × 10^−2^ M	11.9	6	this work
MNP@PD_100_/Au-Ag-Pt/IO	3 × 10^−4^ M	0.2 mg	1 × 10^−2^ M	5.4	12	this work
MNP@PD_60_/Au/IO	3 × 10^−4^ M	0.2 mg	1 × 10^−2^ M	5.3	15	this work
MNP@PD_60_/Au	3 × 10^−4^ M	0.2 mg	1 × 10^−2^ M	1.3	60	this work
PD particles@Au	0.8 × 10^−4^ M	2 mg	1 × 10^−1^ M	3.6	14	[53]
MNP@SiO_2_-Au@mSiO_2_	5 × 10^−3^ M	3 mg	2 × 10^−1^ M	3.3	18	[54]
MNP@SiO_2_-PEI-AgNPs	1.2 × 10^−4^ M	2 mg	5 × 10^−3^ M	1.2	40	[55]
PD membrane@Au	2.0 × 10^−3^ M	- ^a^	6 × 10^−2^ M	4.1	16	[56]
Au/MNP@PDA	1.0 × 10^−4^ M	0.1 mg	1 × 10^−1^ M	6.7	12.5	[57]

^a^ No data presented.

## Data Availability

Data are available upon request by contact with the corresponding author.

## Supplementary Materials

The following supporting information can be downloaded at: https://www.mdpi.com/article/10.3390/nano13061037/s1, Figure S1: SEM and TEM images of MNP@PD_x_ (x = 30 and 100); Figure S2: FT-IR spectra and TGA data of MNP@PD_x_ (x = 30, 60 and 100); Figure S3: STEM images and the corresponding EDX data of MNP@PD_60_/Au and MNP@PD_60_/Au/IO; Figure S4: STEM images and the corresponding EDX data of MNP@PD_60_/M (M: Au-Ag, Au-Pt and Au-Ag-Pt); Figure S5: STEM images and the corresponding EDX data of MNP@PD_60_/M/IO (M: Au-Ag, Au-Pt and Au-Ag-Pt); Figure S6: SEM image of MNP@PD_60_/IO; Figure S7: Time-dependent UV–vis absorption spectral changes of the 4-NPh reduction in the presence of MNP@PD_60_/M/IO (M: Au-Ag, Au-Pt and Au-Ag-Pt); Figure S8: Time-dependent UV-vis absorption spectral changes of the 4-NPh reduction in the presence of MNP@PD_x_/Au-Ag-Pt/IO (x = 30, 60 and 100); Figure S9: Plot of -ln(*C*_t_/*C*_0_) versus time (*t*) for the reduction of 4-NPh with MNP@PD_x_/Au-Ag-Pt/IO (x = 60 and 100); Figure S10: Catalytic reduction rates of 4-NPh with 4-APh as a function of the n reduction cycles by MNP@PD_x_/Au-Ag-Pt/IO (x = 60 and 100).
