# Supplementary material for: Magnetic Iron Oxide Nanoneedles with Hierarchical Structure for Controllable Catalytic Activity of 4-Nitrophenol Reduction
Source: Nanomaterials (Basel). 2023 Mar 13;13(6):1037. doi: 10.3390/nano13061037 (PMC10054365; doi:10.3390/nano13061037)
Supplement: Supplementary file 1 [file nanomaterials-13-01037-s001.zip › nanomaterials-2277234-supplementary.pdf]

Supplementary Materials

Magnetic Iron Oxide Nanoneedles with Hierarchical Structure for Controllable Catalytic Activity of 4-Nitrophenol Reduction

Hyokyung Jeon [1] and Ha-Jin Lee [2,*]

[1] Western Seoul Center, Korea Basic Science Institute, 150 Bugahyun-ro, Seoudaemun-gu, Seoul 03759, Republic of Korea
[2] Division of Chemistry and Bio-Environmental Sciences, Seoul Women's University, 621 Hwarangro, Nowon-gu, Seoul 01797, Republic of Korea
* Correspondence: hajinlee@swu.ac.kr; Tel.: +82-2-970-5654

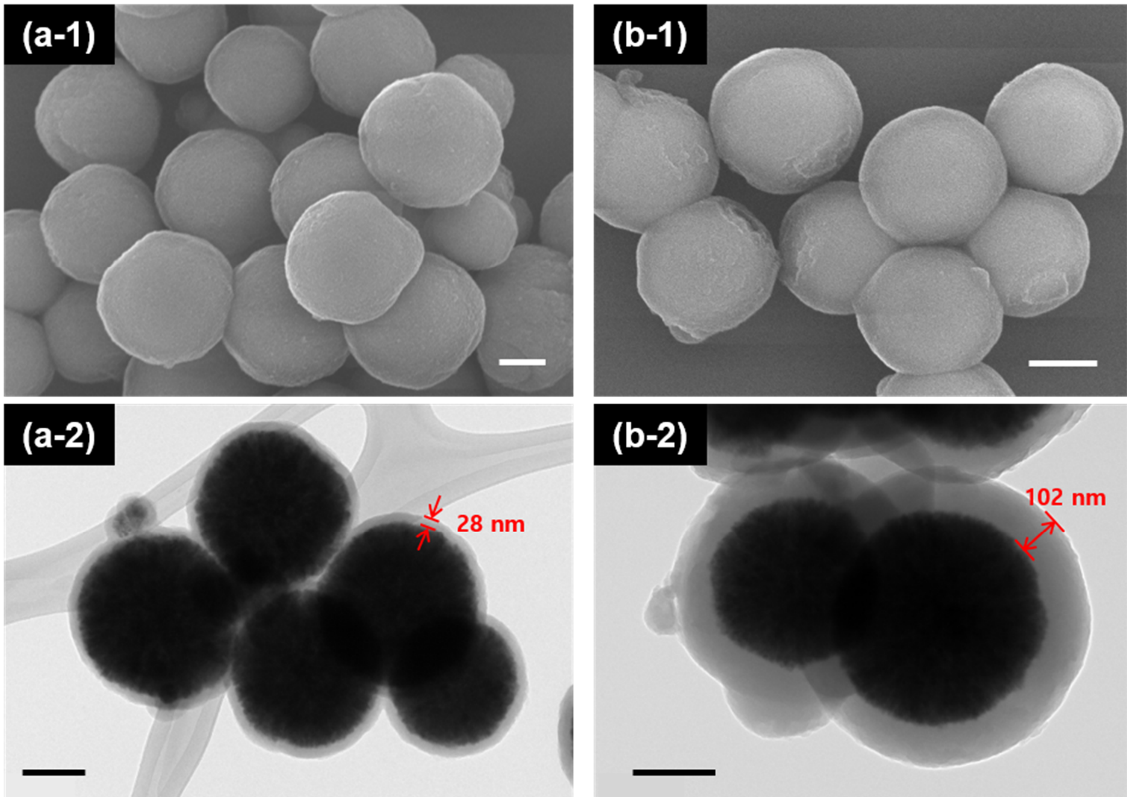

Figure S1. SEM and TEM images of (a) MNP@PD$_{30}$ and (b) MNP@PD$_{100}$. All scale bars represent 200 nm.

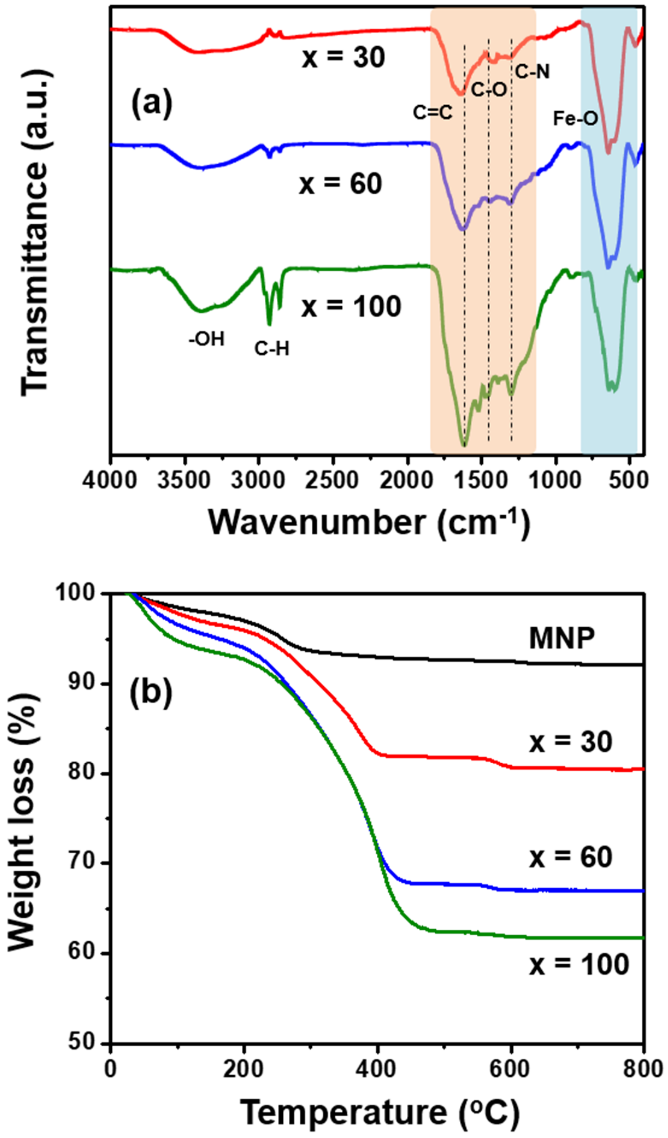

Figure S2. (a) FT-IR spectra and (b) TGA data of $MNP@PD_x$ (x=30, 60 and 100).

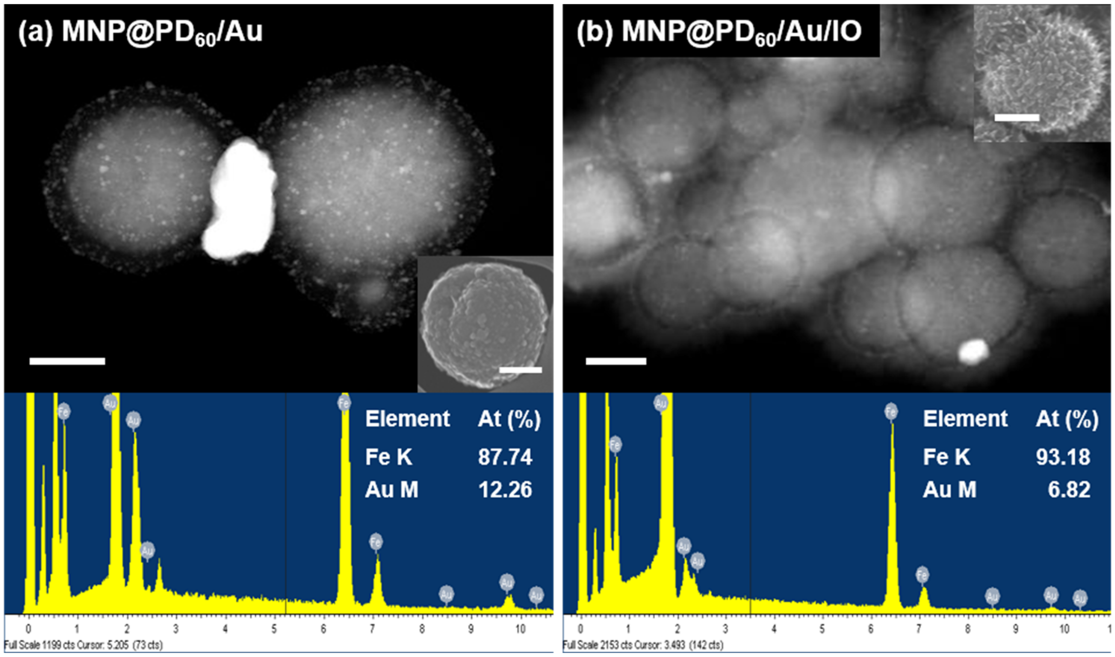

Figure S3. STEM images and the corresponding EDX data of (a) MNP@PD_{60}/Au and (b) MNP@PD_{60}/Au/IO. Insets of the STEM images are the corresponding SEM images, and all scale bars represent 200 nm.

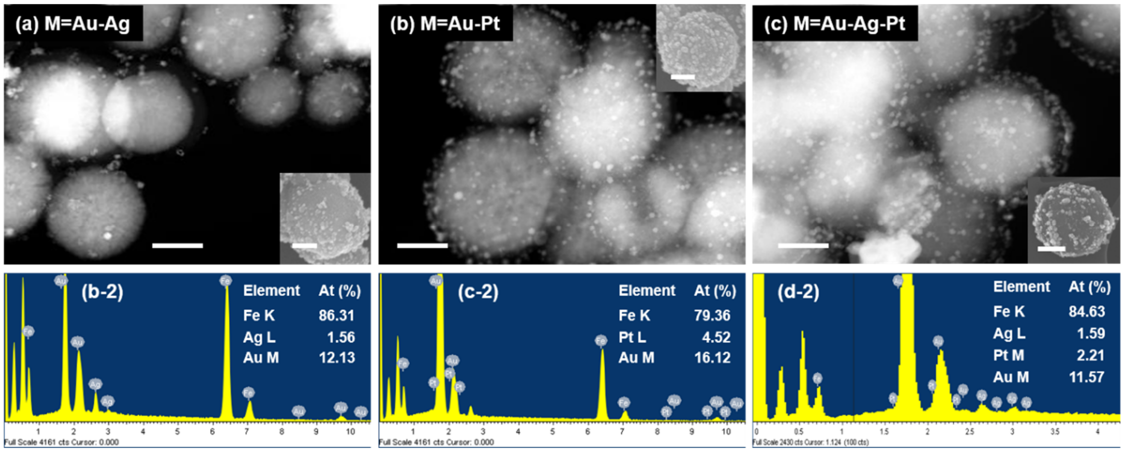

Figure S4. STEM images and the corresponding EDX data of MNP@PD_{60}/M, where M is (a) Au-Ag, (b) Au-Pt and (c) Au-Ag-Pt. Insets of the TEM images are the corresponding SEM images, and all scale bars represent 200 nm.

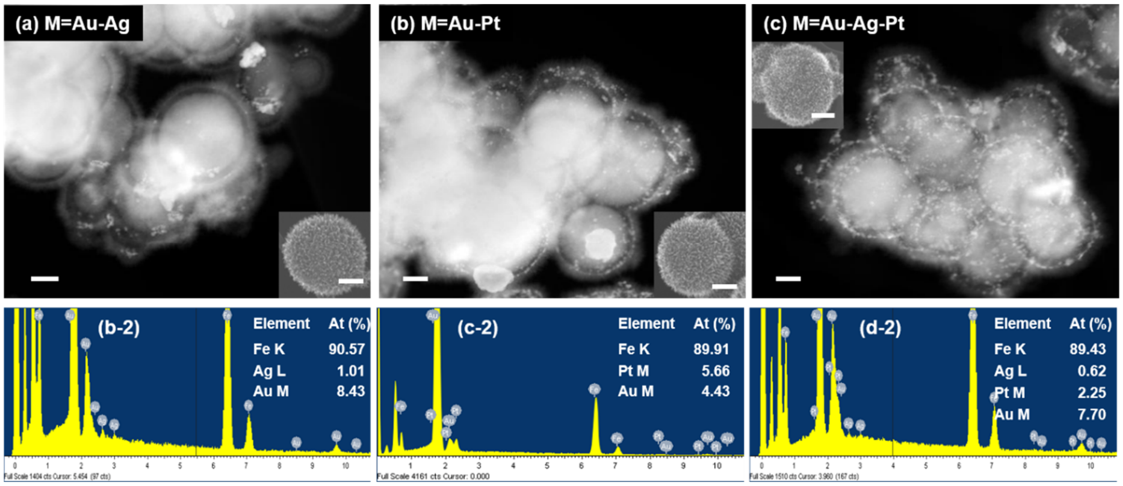

Figure S5. STEM images and the corresponding EDX data of MNP@PD_{60}/M/IO, where M is (a) Au-Ag, (b) Au-Pt and (c) Au-Ag-Pt. Insets of the TEM images are the corresponding SEM images, and all scale bars represent 200 nm.

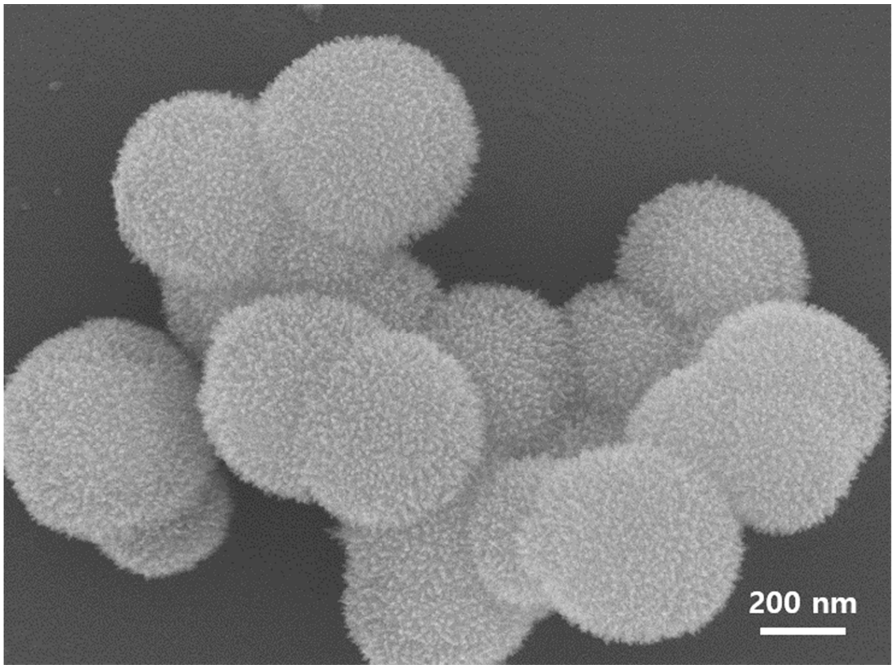

Figure S6. SEM image of MNP@PD_{60}/IO.

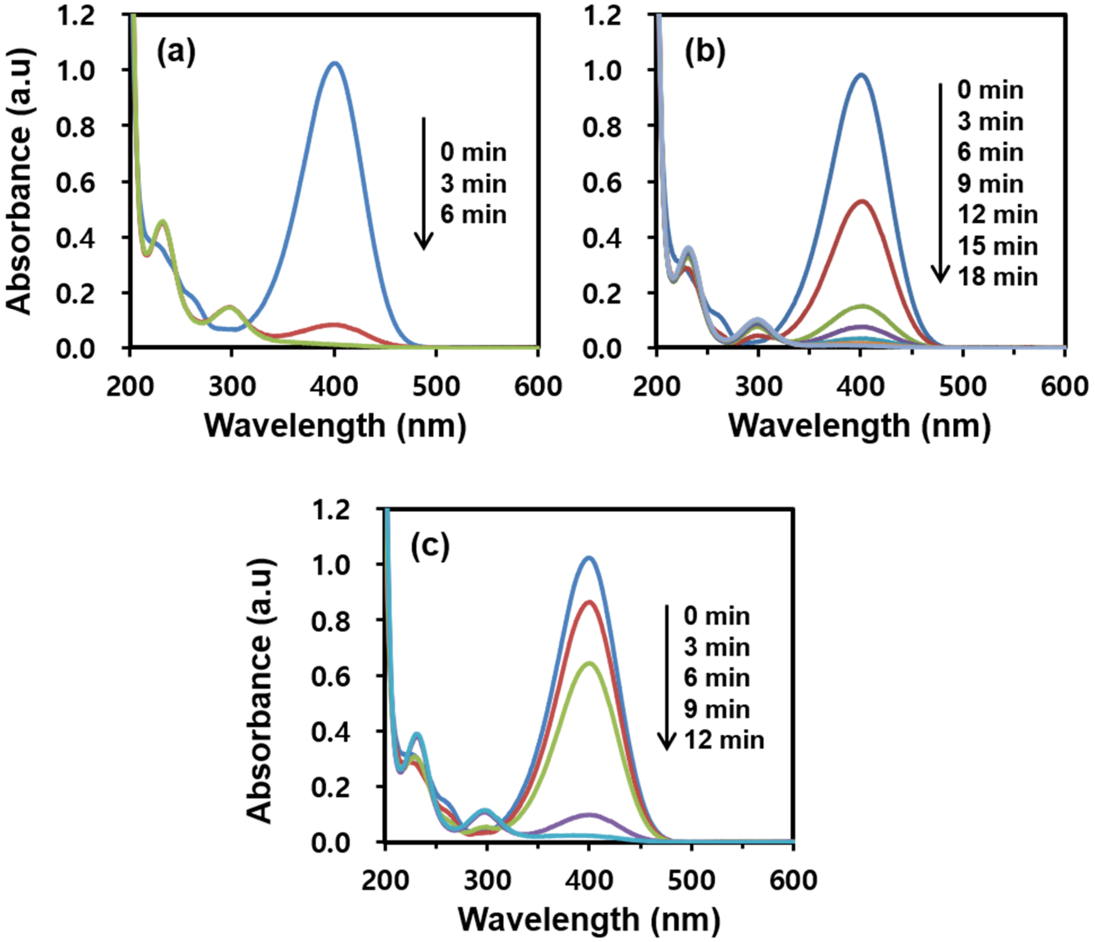

Figure S7. Time-dependent UV–vis absorption spectral changes of the 4-NPh reduction in the presence of MNP@PD$_{60}$/M/IO, where M is (a) Au-Ag-Pt, (b) Au-Pt and (c) Au-Ag.

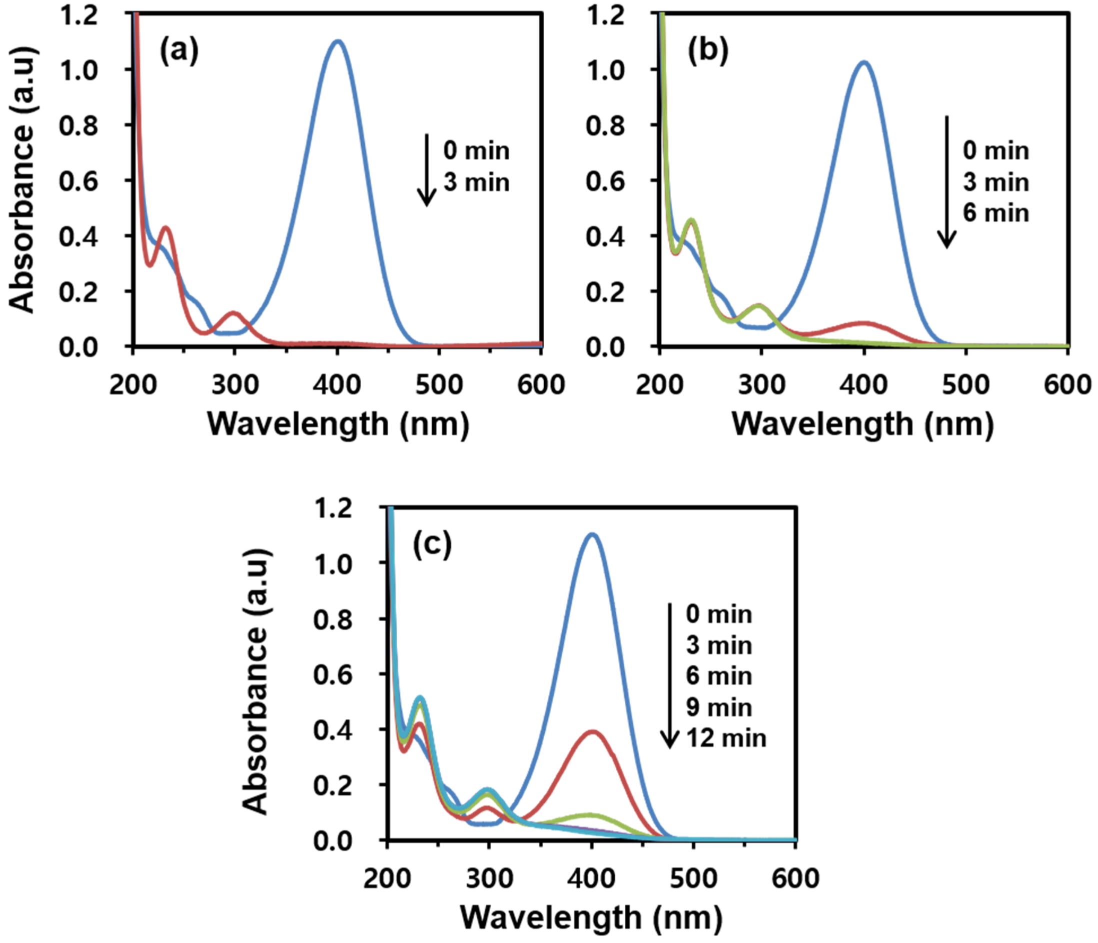

Figure S8. Time-dependent UV-vis absorption spectral changes of the 4-NPh reduction in the presence of MNP@PD_x/Au-Ag-Pt/IO: (a) x=30, (b) x=60 and (c) x=100.

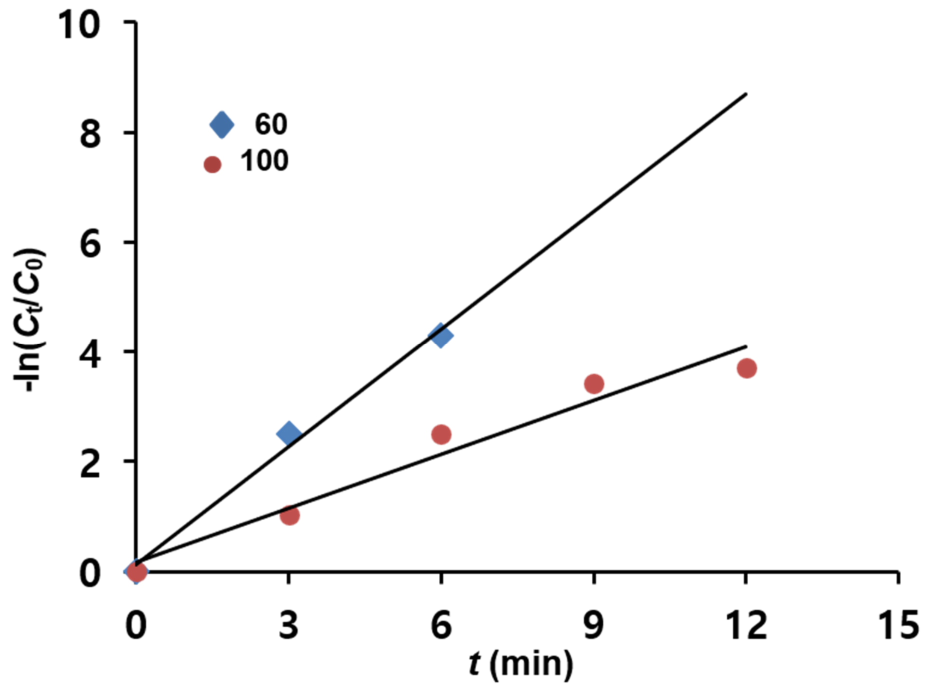

Figure S9. Plot of -ln(C_t/C_0) versus time (t) for the reduction of 4-NPh with MNP@PD_x/Au-Ag-Pt /IO (x=60 and 100).

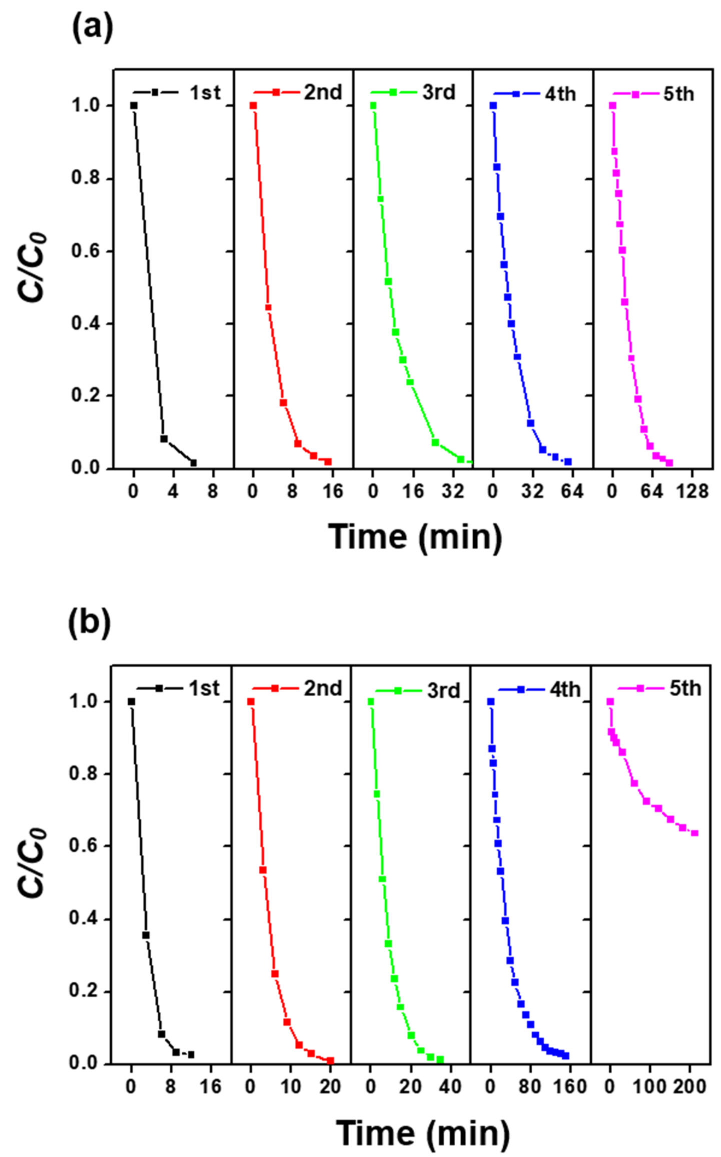

Figure S10. Catalytic reduction rates of 4-NPh with 4-APh as a function of the n reduction cycles by MNP@PD_x/Au-Ag-Pt /IO (x=60 and 100).
